# Elimination Behavior of Shelter Dogs Housed in Double Compartment Kennels

**DOI:** 10.1371/journal.pone.0096254

**Published:** 2014-05-13

**Authors:** Denae Wagner, Sandra Newbury, Philip Kass, Kate Hurley

**Affiliations:** The Koret Shelter Medicine Program, School of Veterinary Medicine, University of California Davis, Davis, California, United States of America; The Department of Population Health and Reproduction, School of Veterinary Medicine, University of California Davis, Davis, California, United States of America; Institut Pluridisciplinaire Hubert Curien, France

## Abstract

For animals in confinement housing the housing structure has tremendous potential to impact well being. Dogs in animal shelters are often housed in one of two types of confinement housing – single kennels and rooms or double compartment kennels and rooms most often separated by a guillotine door. This study examines the effect of housing on the location of elimination behavior in dogs housed in double compartment kennels were the majority of the dogs were walked daily. One side of the kennel contained the food, water and bed and the other side was empty and available except during cleaning time. Location of urination and defecation was observed daily for 579 dogs housed in indoor double compartment kennels for a total of 4440 days of observation. There were 1856 days (41.9%) when no elimination was noted in the kennel. Feces, urine or both were observed in the kennel on 2584 days (58.1%). When elimination occurred in the kennel the probability of fecal elimination on the opposite side of the bed/food/water was 72.5% (95% CI 69.05% to 75.69%). The probability of urination on the opposite side of the bed/food/water was 77.4% (95% CI 74.33% to 80.07%). This study demonstrates the strong preference of dogs to eliminate away from the area where they eat, drink and sleep. Double compartment housing not only allows this – it allows staff the ability to provide safe, efficient, humane daily care and confers the added benefits of reducing risks for disease transmission for the individual dog as well as the population.

## Introduction

There is more to housing than placing an animal in a cage or kennel and simply providing a secure place of confinement. The expectations of confinement housing are that animal health and behavioral well-being are maintained (or improved) and that daily care of each animal can be efficiently and safely provided [1]. For shelter animals, the requirements are even more rigorous: housing must address the needs for viewing by the public for reclaim of lost pets and present animals in such way that an animal’s chance for adoption is maximized.

Type of housing, including cage size, location, and interior set-up, has been linked to stress, health, and chances for adoption in shelter cats [2]–[6]. Although data on environmental enrichment and management of dogs in animal shelters and research facilities are commonly reported, limited research has been done specifically examining the effect of housing type on these parameters for dogs, in particular the effect of the two most common types of confinement housing for individual dogs in North American shelters: single compartment and double compartment kennels or cages.

Each type of confinement housing for shelter dogs has some advantages and limitations. Single compartment cages or kennels save space, and are sometimes stacked on top of one another for further space-saving purposes. These are often used in holding areas of shelters, especially for smaller dogs and puppies. Single rooms are more spacious and commonly used in adoption areas. These are sometimes termed “real life rooms” and can be equipped with furniture to present the dog in a more home-like context. Glass fronts and closed doors allow presentation of dogs for adoption with a minimum of noise and smell. Regardless of relative size, the single compartment unit confines the dog to one living space. Unless the dog is removed to another location at sufficiently frequent intervals, this single space is where the daily activities of eating, drinking, sleeping, ambulating, urination and defecation will occur.

A double compartment housing unit provides dogs with access to two spaces that are separated by a door. These are most commonly two kennels connected by a guillotine type door - either back-to-back or side-to-side. In the case of double compartment rooms, access is provided to a second area (another room, indoor kennel, outdoor kennel, etc.) also with some type of door in between. To preserve the double compartment functionality, access to both sides is allowed for most of the day providing dogs choice in use of both sides of the housing unit.

The obvious limitation of double compartment kennels, cages or rooms is the greater space requirements imposed. However, this type of housing also confers some significant potential advantages. Double compartment housing permits care of the dog without removal from its housing unit, reducing stress in dogs unfamiliar with handling and reducing risk of disease transmission between dogs as well as the risk of bites or injury to the handler, especially when caring for dogs recently admitted to a shelter with unknown health and behavioral status. Cleaning without needing to remove the dog can save substantial time for daily care, offsetting some of the cost and space savings associated with single compartment units. Finally, double compartment housing can provide for the physical separation of functional areas of the housing unit, e.g. for food, water, bedding separated from an area for elimination.

Elimination (urination and defecation) is one of the fundamental biological activities of any species. In addition, there is great significance in the dispersal of feces and urine in the lives of some animals that occurs over and above the simple elimination of waste [7]. The importance of this for dogs was recognized nearly a century ago when the elimination behavior of dogs was described, leading to the suggestion that feces and urine disposal in this species is a means of territory demarcation [8]–[10]. Distribution of feces away from the “den” or primary living quarters may also have implications for reduction of disease transmission [11], and as such may represent a strongly selected behavior in canines. Multiple studies were instrumental in documenting the strong preference of dogs for a localized defecation area [12]–[14]. This preference develops early in life and was first described in puppies as young as one month of age [13]. Even in the absence of specific housebreaking training, the preference for a localized defecation area has been observed in adult laboratory dogs, and in one study adult dogs that had access to an outside exercise area only through an open window always chose to defecate outdoors during a 30 day observation period [12].

Given the importance of elimination location for dogs, providing housing that supports the dog’s natural preference may be a significant factor in ensuring behavioral health and alleviating stress. Our hypothesis was 1. When dogs are housed in double compartment housing units, dogs would exhibit a preference for a localized area for fecal and urine elimination. 2. The side of the housing unit away from the location of the bed, food and water would be preferentially used for elimination. This study examines the location where dogs defecated and urinated within a double compartment indoor/indoor kennel and provides further evidence that fecal and urine elimination behavior in dogs is not a random event.

## Materials and Methods

Ethics Statement: no permits were required for this study. This study was limited to observation/documentation of the elimination behavior of dogs housed in an animal shelter. Both shelters gave permission for the study to occur on their site. No changes were made to the housing or handling practices of the participating shelters and no animal suffering occurred as a result of this study.

Dogs from two shelters were enrolled in this study, one in Wisconsin (data collected from March 2011 through June of 2011) and one in Virginia (data collected in December 2010). All dogs housed in double compartment kennel housing areas were enrolled. These areas included holding, medical/isolation and quarantine at both shelters, and in addition adoption housing at the Wisconsin shelter. All double compartment kennels in the study were fully indoors, with the compartments arranged front to back with a pass through that could be closed via a guillotine door. Most kennels were 4′ wide × 12′ long with a guillotine door located at the center making each side of the kennel approximately 6′ long. (Fig. S1).

Any dog entering the shelter and housed in a double compartment kennel during the study period was included in the study. The age of each dog was collected from the owner or estimated at the time of intake by shelter staff. Dogs ranged in age from 2 months to 14 years. Dogs received a recording sheet upon placement in the kennel housing area. For each dog, the location of fecal and urine elimination within the kennel was documented daily by shelter staff prior to morning cleaning. Elimination location within the kennel was reported as: no feces or urine in the run, feces on side with bed, feces on side with no bed, feces on both sides, no feces in run, urine on side with bed, urine on side with no bed, urine on both sides and no urine in run. Elimination location was recorded for up to the first 12 days of shelter stay. Both shelters accepted stray and owner surrendered dogs as well as dogs transferred from other shelters.

Dogs in both shelters were part of a robust dog-walking program primarily overseen by volunteers. In general starting approximately 3 days post intake, most dogs were eligible for walking by the volunteer programs and most dogs were walked 2–3x daily. Dogs not participating in the walking program were dogs in their first 3 days of holding, sick dogs housed in isolation and dogs in quarantine.

Individual daily walking activity was not recorded. Probabilities for location of fecal and urine elimination were calculated with variance estimation using a robust method to account for multiple measurements taken on the same dogs [15].

## Results

The location of defecation and urination on the kennel floor was recorded for 579 dogs housed in indoor double compartment kennels at the two study shelters (32 from the Virginia shelter and 547 from the Wisconsin shelter). The average age of the dogs was 2.9 years. The number of observation days per dog ranged from 1 to 12 days (average 7.7 days) for a total of 4440 days of observation. The data collected came from dogs housed in the following areas: 563 days in adoption (12.67%), 2909 days in holding (65.47%), 78 days in isolation (1.75%), 692 days in medical (15.57%), and 197 days in quarantine (4.43%). The type and location of elimination is shown in Table 1.

**Table 1:** Elimination occurrence and location for dogs housed within double compartment indoor kennels in an animal shelter.

Shelter	Numberof dogs	DaysObserved	No fecesor urine in run	Feces onside with bed	Feces onside with no bed	Feces onboth sides	No fecesin run	Urine onside with bed	Urine onside with no bed	Urine onboth sides	NoUrine in run
Virginia	32	246	50	1	138	23	33	6	157	12	20
Wisconsin	547	4194	1806	252	1433	319	373	244	1618	258	245
Total	579	4440	1856	253	1571	342	406	250	1775	270	265

**Figure 1:**
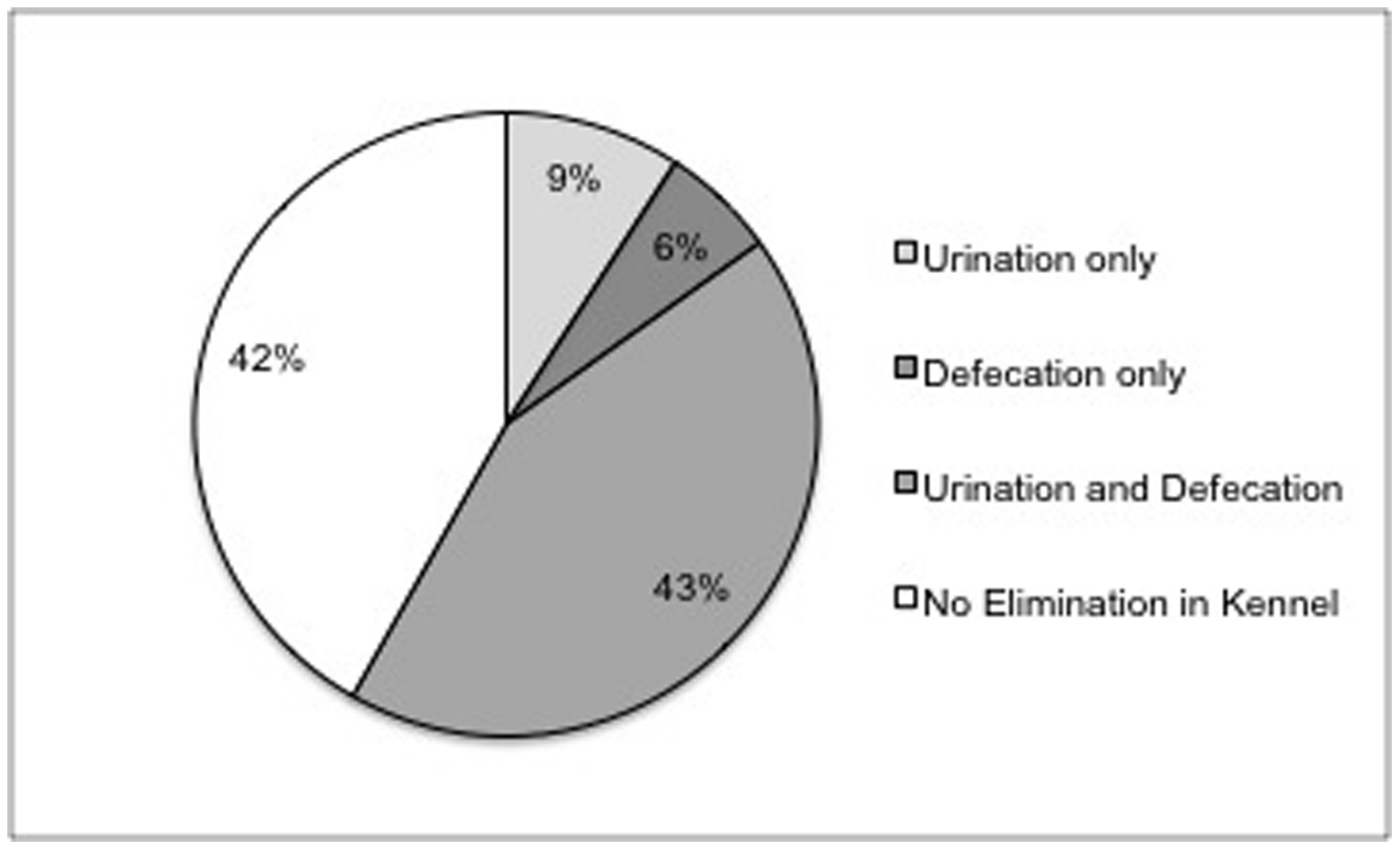
Fecal and/or urine elimination within the kennel for dogs walked 2–3x daily and housed in an animal shelter. Dogs did not eliminate in the kennel 41.9% of the time in this study which can be accounted for by daily walking programs occurring in each shelter. Walking programs allow dogs the opportunity to eliminate outside the housing environment, however a majority, 58.1%, of dogs still eliminated within their housing unit indicating that the dogs elimination needs may not be completely addressed with daily walking programs. *(Note: All dogs enrolled in the study were included. Dogs that were sick, in quarantine or in their first three days of their hold were not walked).*

There were 1856 days (41.9%) where no elimination was noted in the kennel. Feces, urine or both were observed in the kennel on 2584 days (58.1%). (Fig. 1).

**Figure 2:**
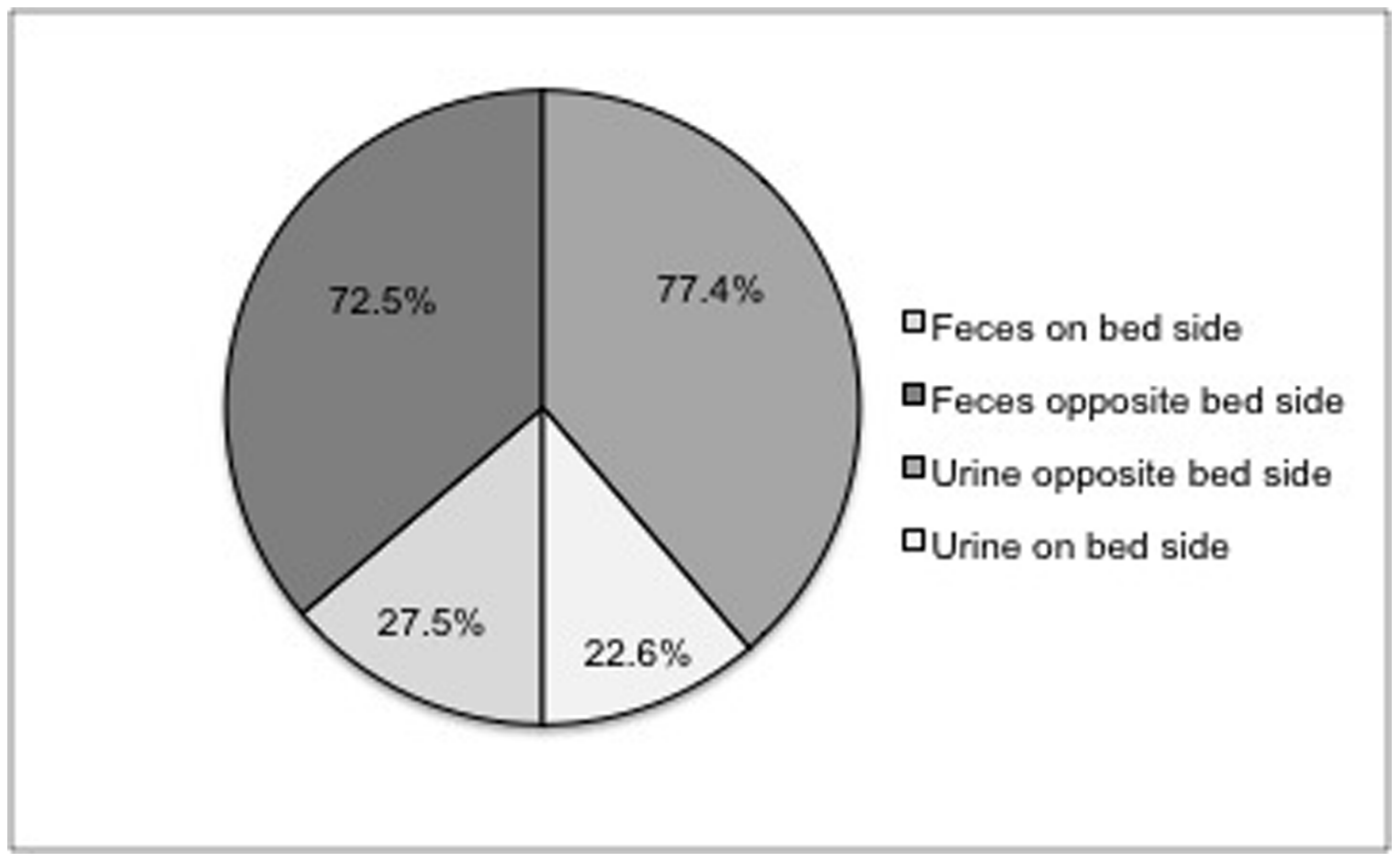
Location of elimination of feces and urine when elimination occurred within indoor double compartment kennels. When elimination occurred in the kennel the probability of fecal elimination on the opposite side of the bed/food/water was 72.5% (95% CI 69.05% to 75.69%). The probability of urination on the opposite side of the bed/food/water was 77.33% (95% CI 74.33% to 80.07%). This data indicates a strong preference by the dogs to urinate and defecate in the opposite compartment from where their food, water and bed were located. *(Note: All dogs that eliminated in their kennel were included. Dogs that were sick, quarantined or in their first three days of hold were not walked.).*

When elimination occurred in the kennel the probability of fecal elimination on the opposite side of the bed/food/water was 72.5% (95% CI 69.05% to 75.69%). The probability of urination on the opposite side of the bed/food/water was 77.4% (95% CI 74.33% to 80.07%). (Fig. 2).

## Discussion

Confinement housing for dogs is used in a variety of facilities from veterinary schools, and private veterinary practices and hospitals to boarding facilities, breeding facilities, hunting dog owners and animal shelters. The data from this study builds upon what is already known about elimination behaviors in this species and the ideal characteristics of confinement housing.

Dogs in this study demonstrated a very strong tendency not to urinate or defecate on the side of a double compartment kennel containing food, water and a bed (the “den” area): no elimination took place in the kennel over 40% of the time, and when elimination did take place within the kennel, it was on the side opposite the den over 70% of the time. Walking programs are often proposed as a solution to allow dogs confined in single compartment housing to avoid soiling their quarters. However, the prior housebreaking habits of a confined dog are often unknown. Shelter dogs may never have been housebroken, and may not know to take advantage of a brief opportunity to eliminate when they are removed from the kennel. Even a pet dog in a boarding kennel or veterinary clinic may be accustomed to use of a dog door rather than eliminating at specific, relatively brief intervals of outdoor access. Dogs that have been harshly punished for eliminating in the house may even develop an aversion to elimination in the presence of an observer. Finally, walking programs may not always be coordinated with feeding programs or may not take place at sufficiently short intervals to allow a dog to hold its urine and feces between walks. All these may explain why elimination occurred in the kennel over half the time (58.1%) even though a walking program was in place for most of the dogs at both shelters. These data suggest that daily walking for elimination is important but not a replacement for housing designed such that elimination can occur away from the daily activities of eating/drinking and resting.

The significant preference for dogs to eliminate away from the den, when elimination did take place in the kennel, suggests that providing this opportunity is important to meet the behavioral preference of dogs.

While it is not known whether failing to meet the behavioral preference to eliminate away from the den induces significant stress, it is reasonable to speculate that some stress could occur when such a strongly preferred behavior is prevented. For shelter dogs the stakes may be even higher: the preference to avoid soiling the sleeping quarters is the foundation of “crate training”, a commonly recommended method of housebreaking. Problems with house breaking behavior and house training for dogs are documented key components of pet retention and adoption success. [16], [17] Forcing a dog to habituate to soiling its sleeping quarters may reduce the effectiveness of this important training tool.

This study was performed as a pilot study and does not address all the possible variables that may affect location of urination and defecation in confinement housing- primarily the roles of housing type prior to shelter intake, sex, kennel size, previous occupants in the housing unit, disinfectant use and the housebreaking history of the dog. Additionally because the food, water and bed were all located on the same side of the double compartment kennel it is not known whether their individual location has more or less of an effect on the outcome of the location of urination and defecation in the housing unit.

## Conclusion

While the exact ramifications of double versus single compartment confinement housing on stress and housebreaking habits of dogs from a variety of backgrounds remains to be elucidated, this study clearly demonstrated the strong preference of dogs to eliminate away from the area where they eat, drink and sleep.

Providing double compartment housing not only allows this – it allows staff to provide safe, efficient, humane daily care and confers the added benefits of reducing risks for disease transmission for the individual dog as well as the population. Double compartment housing can be provided in the context of either a run or a room, with compartments either both indoors, or one indoors and one out. Given the substantial potential advantages for animal well being, staff safety and efficiency of care, the positive attributes of double compartment housing for dogs may outweigh the additional space required in many situations.

## Supporting Information

Figure S1 **Example of double-compartment housing at the Wisconsin shelter.**
(TIFF)
