# Supplementary figures and images for: Elimination Behavior of Shelter Dogs Housed in Double Compartment Kennels
Source: PLoS One. 2014 May 13;9(5):e96254. doi: 10.1371/journal.pone.0096254 (PMC4019474; doi:10.1371/journal.pone.0096254)

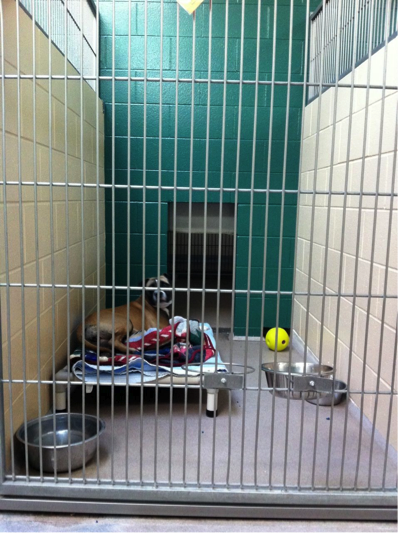

Supplement: Figure S1 — Example of double-compartment housing at the Wisconsin shelter. (TIFF) [file pone.0096254.s001.tiff]
